# Exploring the SARS-CoV-2 virus-host-drug interactome for drug repurposing

**DOI:** 10.1038/s41467-020-17189-2

**Published:** 2020-07-14

**Authors:** Sepideh Sadegh, Julian Matschinske, David B. Blumenthal, Gihanna Galindez, Tim Kacprowski, Markus List, Reza Nasirigerdeh, Mhaned Oubounyt, Andreas Pichlmair, Tim Daniel Rose, Marisol Salgado-Albarrán, Julian Späth, Alexey Stukalov, Nina K. Wenke, Kevin Yuan, Josch K. Pauling, Jan Baumbach

**Affiliations:** 1 Chair of Experimental Bioinformatics, TUM School of Life Sciences, Technical University of Munich, München, Germany; 2 Institute of Virology, TUM School of Medicine, Technical University of Munich, München, Germany; 3 LipiTUM, Chair of Experimental Bioinformatics, TUM School of Life Sciences, Technical University of Munich, München, Germany; 4 Natural Sciences Department, Universidad Autónoma Metropolitana-Cuajimalpa (UAM-C), 05300 Mexico City, Mexico; 5 Computational Biomedicine Lab, Department of Mathematics and Computer Science, University of Southern Denmark, Odense, Denmark

**Keywords:** Network topology, Target identification, Software, Viral infection

## Abstract

Coronavirus Disease-2019 (COVID-19) is an infectious disease caused by the SARS-CoV-2 virus. Various studies exist about the molecular mechanisms of viral infection. However, such information is spread across many publications and it is very time-consuming to integrate, and exploit. We develop CoVex, an interactive online platform for SARS-CoV-2 host interactome exploration and drug (target) identification. CoVex integrates virus-human protein interactions, human protein-protein interactions, and drug-target interactions. It allows visual exploration of the virus-host interactome and implements systems medicine algorithms for network-based prediction of drug candidates. Thus, CoVex is a resource to understand molecular mechanisms of pathogenicity and to prioritize candidate therapeutics. We investigate recent hypotheses on a systems biology level to explore mechanistic virus life cycle drivers, and to extract drug repurposing candidates. CoVex renders COVID-19 drug research systems-medicine-ready by giving the scientific community direct access to network medicine algorithms. It is available at https://exbio.wzw.tum.de/covex/.

## Abstract (web summary)

Information developed to understand the molecular mechanisms of SARS-CoV-2 infection for predicting drug repurposing candidates is time-consuming to integrate and explore. Here, the authors develop an interactive online platform for virus-host interactome exploration and drug (target) identification.

## Introduction

Coronavirus Disease-2019 (COVID-19) is an infectious disease caused by SARS-CoV-2 (severe acute respiratory syndrome coronavirus 2). It was first identified in Wuhan, China and has spread causing an ongoing pandemic^1^ with globally 2.4 million confirmed cases and 167 thousand deaths as of April 20, 2020.

Our insights into SARS-CoV-2 infection mechanisms are limited and clinical therapy has largely focused on treating critical symptoms. Therefore, the current pandemic requires fast and freely accessible knowledge to accelerate the development of vaccines, treatments, and diagnostic tests. Research data have been collected in several online platforms, such as the COVID-19 Open Research Dataset and the Dimensions COVID-19 collection^2,3^. In addition, existing databases that collect virus information have responded by integrating new SARS-CoV-2 research^4,5^.

As vaccine and drug development may take years, drug repurposing is a potent approach that offers new therapeutic options through the identification of alternative uses of already approved drugs^6^. These drugs have previously undergone clinical and safety trials and, hence, accelerate drug development timelines from a decade to a few years or months. Due to the COVID-19 pandemic, numerous research groups around the world have been joining their efforts to identify drugs that can be repurposed to effectively treat COVID-19. Numerous drugs are already part of clinical trials, including Remdesivir (a less effective ebola drug), Chloroquine, Hydroxychloroquine (antimalarial drugs), Tocilizumab (rheumatoid arthritis drug), Favipiravir (influenza drug), and Kaletra (a combination of Lopinavir and Ritonavir for treating human immunodeficiency virus HIV-1)^7^.

**Fig. 1:**
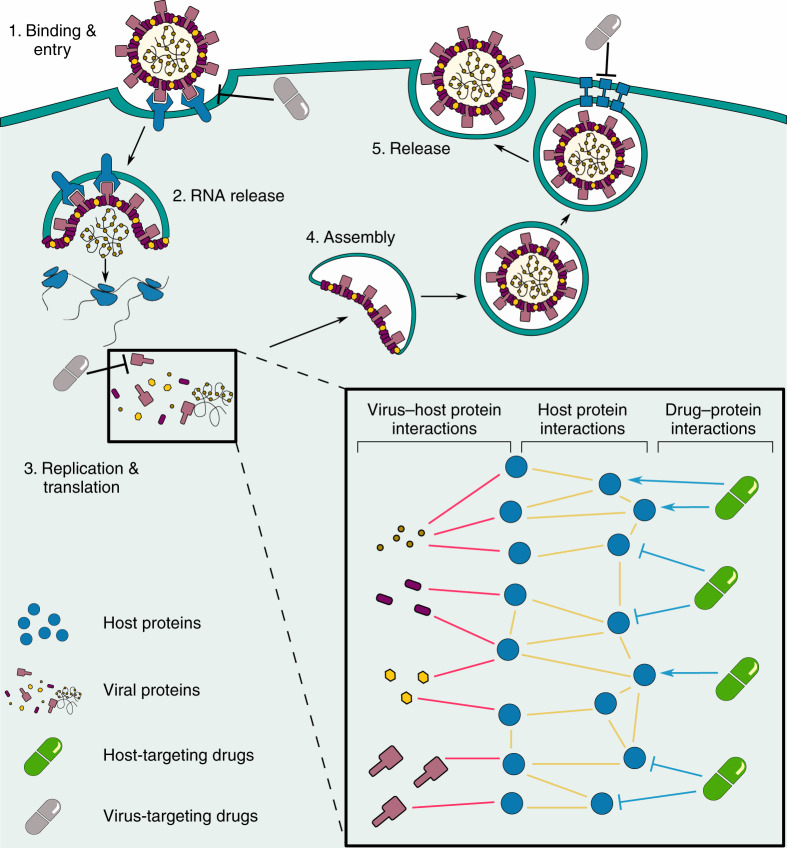
The SARS-CoV-2 life cycle and the CoVex systems medicine approach of drug repurposing. Most antiviral drugs (gray drugs) target the virus proteins or their direct host interactor proteins to inhibit different stages of the viral life cycle. Our rationale, however, is that viral interactions with human host proteins have a cascading effect to hijack and control key proteins necessary for the virus’ life cycle. We aim to identify repurposable drug candidates (green drugs) targeting these key host modulators to interfere with virus replication and disease progression following infection. Besides an increased antiviral drug repertoire, targeting host proteins would make it more difficult for the virus (population) to develop resistance mutations.

Computational systems and network medicine approaches offer a methodological toolbox required to understand molecular virus–host–drug mechanisms and to predict novel drug targets to attack them^8,9^. Few studies on these mechanisms in SARS-CoV-2 exist. Gordon et al.^10^ applied affinity purification-mass spectrometry (AP-MS) to reconstruct the SARS-CoV-2-human protein–protein interaction (PPI) network and subsequently employed a chemoinformatics approach to identify potential drugs for repurposing. The data generated from that study is a major advancement in understanding SARS-CoV-2 infection. However, to identify drug candidates, the study mainly considered the direct interactors of the human proteins as putative targets and thus did not take into account the network context of the human interactome. However, viral interactions with human proteins have cascading effects in the human interactome, where key proteins necessary for the viral replication cycle are only indirectly affected. Therefore, downstream host proteins may be additional promising targets for therapeutic intervention, but require thorough data integration and mining to be identified (see Supplementary Methods for details). Figure 1 illustrates the concept of systems medicine-based drug repurposing specifically for SARS-CoV-2.

Gysi et al.^11^ integrated the experimentally validated SARS-CoV-2 virus–host interactions with the human interactome and investigated comorbidity and differences of virus–host interactions across 56 tissues. Furthermore, network medicine analysis was applied to compile a list of drug repurposing candidates that target also indirectly affected proteins in the human interactome. However, the combined number of virus–host, host–host, and drug–target interactions goes into the millions such that purely algorithmic approaches to discovering new drug targets and drug repurposing candidates produces a large number of results, many of which lack mechanistic specificity and, hence, are not useful. Thus, to make their results accessible, Gysi et al.^11^ worked closely together with clinical experts to narrow down the number of predicted repurposable drugs.

In order to allow for the interactive integration of expert knowledge about virus replication, immune-related biological processes, or drug mechanisms, we developed the interactive systems and network medicine platform CoVex (CoronaVirus Explorer). It integrates experimental virus–human interaction data for SARS-CoV-2 and SARS-CoV-1 with the human interactome as well as drug information to predict novel drug (target) candidates, and it offers biomedical and clinical researchers’ interactive and user-friendly access to network medicine algorithms for advanced data mining and hypothesis testing. CoVex follows a human-in-the-loop paradigm and provides an intuitive visualization of virus–host interactions, drug targets, and drugs to enable researchers to examine molecular mechanisms that can be targeted using repurposed drugs. CoVex offers two main actions for which several network medicine algorithms are available: Given a list of user-selected human host proteins, viral proteins, or drugs (referred to as seeds), users can (1) search the human interactome for viable drug targets and (2) identify repurposable drug candidates. In a typical workflow, these two actions are combined, that is, starting from a selection of virus or virus-interacting proteins, users mine the interactome for suitable drug targets for which, in turn, suitable drugs are identified. Additionally, users can leverage expert knowledge by uploading a list of proteins or drugs of interest as seeds to guide the analysis. Such seeds could, for instance, be a list of differentially expressed genes (DEGs), a list of proteins related to a molecular mechanism of interest, or a set of drugs known to be effective.

The remainder of this paper is structured as follows: In the “Methods” section, we first describe the datasets and integration strategy used in CoVex. Next, we introduce the rationales of the systems and network medicine algorithms implemented in CoVex, and briefly describe the overall architecture of the platform. In the “Results” section, we show several application examples to illustrate the flexibility and typical use cases of CoVex. Finally, we will discuss opportunities and limitations in using CoVex for COVID-19 research.

CoVex opens up the systems medicine toolbox for the entire infectious disease research community by providing an easy-to-use web tool enriched with data mining algorithms for drug repurposing. This allows specialists from different fields to bring in expert knowledge to identify the most promising drug targets and drug repurposing candidates for developing effective therapies. We would like to stress that the CoVex platform can and will be adopted and extended to allow exploring other viral–host–drug interactomes, for example, with MERS (Middle East respiratory syndrome), Zika, dengue, and influenza viruses, thereby increasing preparedness for similar future events.

## Results

### The CoVex platform

**Fig. 2:**
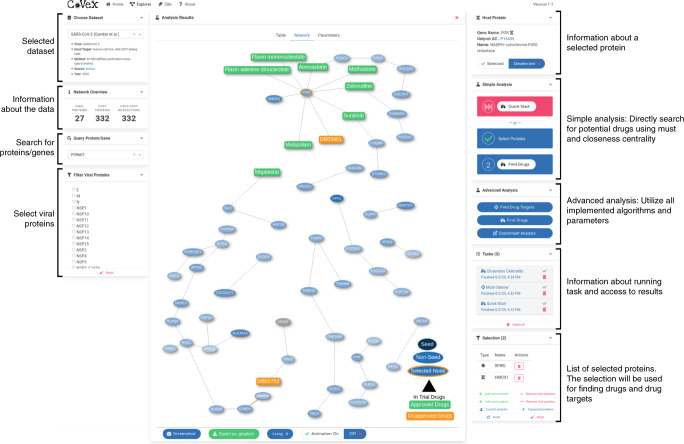
The CoVex online platform. The network view (middle) shows drug candidates (green nodes) that were found using closeness centrality on a set of proteins (blue nodes), which resulted from a multi-Steiner tree computation with all viral proteins as seeds (not shown here). Therefore, drugs targeting these seeds might be able to interrupt the viral life cycle progression. Here we colored nodes based on lung-tissue-specific median gene expression according to GTEx.

**Fig. 3:**
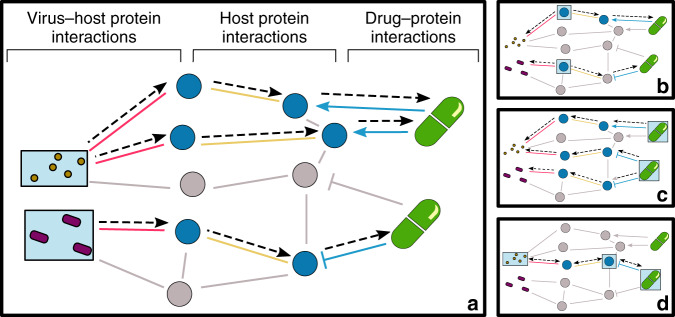
CoVex application scenarios. Depending on the starting hypothesis, dedicated systems medicine algorithms will propagate from selected seeds to connect drugs with viral proteins using host proteins as proxies. Essentially, four different strategies apply: **a** Starting with viral proteins, one can identify drugs targeting host proteins that connect the viral seeds. **b** Starting with a set of proteins of interest as proxies, we identify pathways connecting them to (selected or all) viral proteins. Subsequently, we identify drugs targeting this mechanism. **c** Starting with a set of drugs of interest, one may find pathways to (selected or all) viral proteins extracting a potentially druggable host mechanism. **d** Hypothesis-driven, hybrid approach with seeds in different levels to be connected for druggable mechanism extraction. Boxes with light blue background indicate the typical starting points in the respective application scenario.

The main result is the CoVex platform itself, which renders drug repurposing research systems-medicine-ready. In the following, we first describe how the platform’s user interface (Fig. 2) provides the full feature spectrum of CoVex to clinicians and scientists. Afterwards, we demonstrate the use of CoVex in four different application scenarios starting with four hypotheses and ending with different drug repurposing candidates, as well as a short discussion on how to prioritize them (Fig. 3).

Figure 2 shows the CoVex web interface. To find potential drugs, the “Quick Start” analysis will produce a multi-Steiner tree, which considers all viral proteins as seeds and adds a small number of host proteins to connect them. Subsequently, drugs directly targeting these proteins are selected via closeness centrality. After the computation has finished, a click on the corresponding task opens the analysis results, consisting of a table view of drugs and proteins, a visualization of the protein–protein and drug–protein interactions, and a list of parameters used for the analysis. In the “Simple Analysis” panel, users can select seed proteins manually and search for drugs targeting them. In the “Advanced Analysis” panel, users can choose from a list of network medicine algorithms (see “Methods” and Supplementary Methods for details) to discover drug targets or drug repurposing candidates. Users can either select proteins from the view, upload a custom list of proteins or drugbank ids, or select proteins expressed in a given tissue. An enrichment analysis of the identified drug target proteins may be performed with g:Profiler^12^.

### Application scenarios

The utility of CoVex and its integrated systems medicine approaches is outlined in the following four scenarios. More details on each can be found in the Supplementary Notes.

**Fig. 4:**
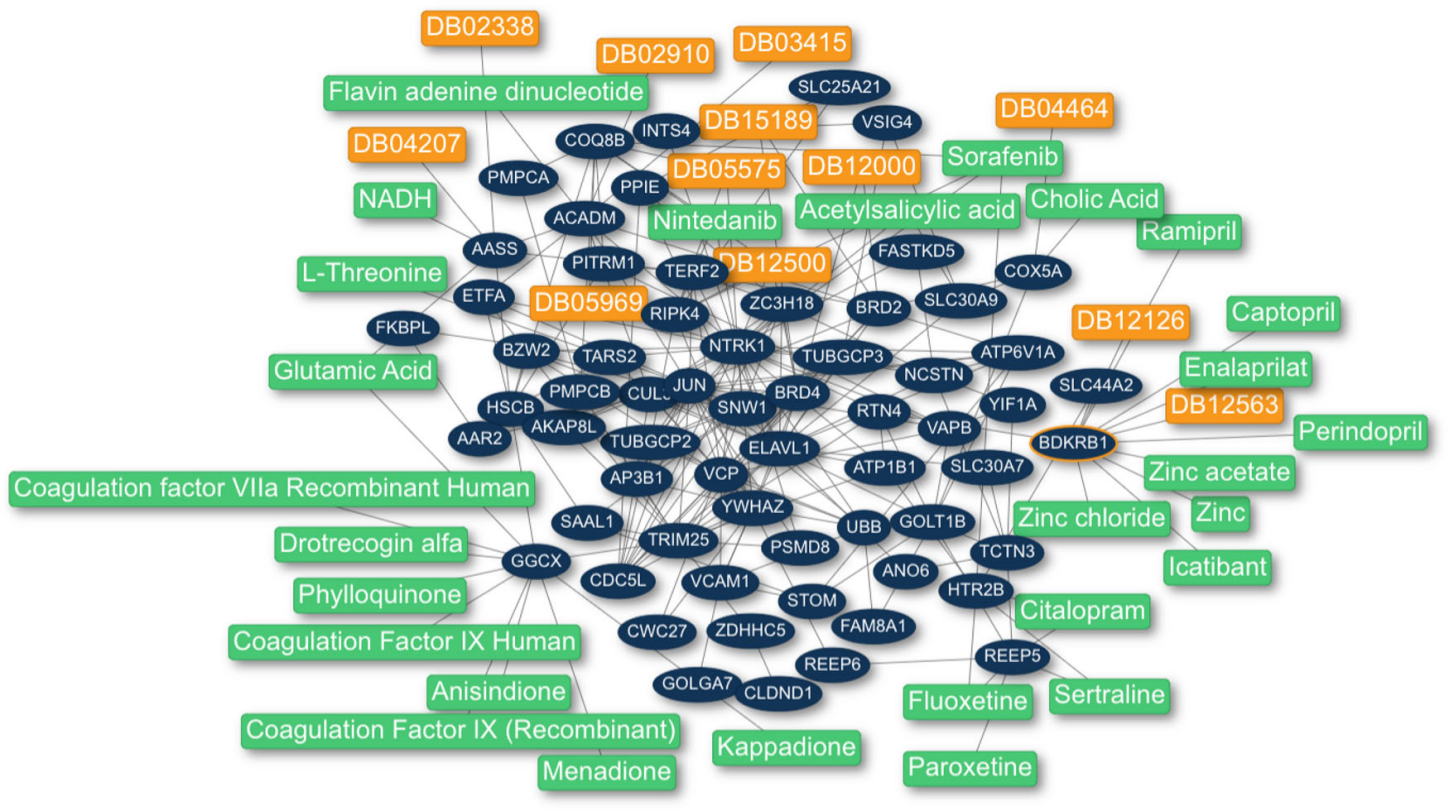
CoVex result network for application scenario a. Drug–protein–protein interaction network obtained using the viral proteins E, M, and Spike with multi-Steiner tree followed by closeness centrality. Blue nodes are protein targets. Green nodes are approved drugs and orange nodes are non-approved drugs. Lines represent the interactions between proteins and drugs. Note that some ACE inhibitor drugs have been identified, such as Ramipril, Captopril, Perindopril, and Enalaprilat targeting the BDKRB1 protein, which are currently being evaluated in clinical trials.

*Scenario a*: Starting from a selection of viral proteins, we use the PPI network to identify the biological mechanism or pathway utilized by the virus. As an example, we consider the viral proteins E, M, and Spike, which constitute the external structure of the virus and thus mediate entry into the host cells during the infection process^13,14^. We select the interactors of these viral proteins reported for SARS-CoV-2 and use the multi-Steiner tree algorithm to uncover the biological pathway involved. The resulting network (Fig. 4) yields 26 new potential drug targets, including the bradykinin receptor B1 (BDKRB1). Subsequently, we use closeness centrality to find drugs affecting this pathway. Notably, we identify six relevant drugs that target BDKRB1: Ramipril, Captopril, Perindopril, and Enalaprilat (approved), which belong to the angiotensin-converting enzyme (ACE) inhibitor class^15^; Icatibant, an antagonist of the bradykinin receptor B2^16^; and bradykinin, a non-approved drug that is degraded by the ACE^17^. Furthermore, to understand the relationship between BDKRB1 and two proteins known to participate in the entry of the virus (angiotensin-converting enzyme 2 (ACE2) and transmembrane protease serine 2)^18^, we use the “custom proteins” option available in CoVex. We found that kininogen 1 and angiotensin proteins connect BDKRB1 with ACE2. These four proteins are functionally related through the renin–angiotensin system, which is targeted by ACE inhibitors (www.wikipathways.org/instance/WP554). In summary, CoVex identifies the protein BDKRB1, which appears to play a role in SARS-CoV-2 host cell entry and can be targeted by several ACE inhibitors widely used in clinical trials to treat COVID-19. It should be noted that the ACE2 protein is not present in the set of seeds used to start the analysis. Nevertheless, CoVex is capable of identifying the pathway and new protein targets functionally related to ACE2 (Fig. 4).

*Scenario b*: Starting from both viral proteins and a list of proteins of interest, we can use CoVex to identify a connecting pathway or biological mechanisms that can be targeted by drugs. In this scenario, we are specifically interested in viral proteins that suppress host immunity and the corresponding host immune response pathways. First, we select the viral proteins ORF7a and ORF3a, which are potentially involved in innate immune response and apoptosis as discussed by Gordon et al.^10^. Next, we compile a list of proteins of interest based on the DEGs from the study by Blanco-Melo et al.^19^ lung epithelial cells were infected with the SARS-CoV-2 virus, leading to altered expression of immunity-related genes to combat the viral infection. We consider DEGs known to be associated with the host pathway involving infection with the herpes simplex virus, another viral pathogen. These genes include *IFIH1*, *OAS1*, *STAT1*, *DDX58*, *OAS2*, *OAS3*, *IRF7*, *EIF2AK2*, *IFIT1*, and *IRF9*. The selected viral proteins and DEGs (converted to Uniprot ids) were used as seeds for the multi-Steiner tree algorithm to extract a potential immune-related mechanism. As expected, the resulting subnetwork reveals that the viral proteins are close to the DEGs in the host PPI network. Closeness centrality analysis assigned a high rank to Tofacitinib and Ruxolitinib, which are currently being assessed in clinical trials. Tofacitinib and Ruxolitinib exert immunomodulatory effects as Janus kinase inhibitors^20,21^. Thus, administration with these drugs may mitigate immune-mediated lung injury and reduce functional deterioration caused by an overamplified host inflammatory response. This could be especially important in later stages of the disease to prevent an overreaction of the body’s immune system and, hence, may further prevent the need for mechanical ventilation in patients suffering from severe COVID-19. Other drugs that target this subnetwork include Masitinib, Erlotinib, and Sorafenib, which could be further examined in downstream analyses. In a similar manner, users may provide a custom list of proteins as seeds to hunt for drugs that can target a putative mechanism of interest.

*Scenario c*: Starting with a set of drugs of interest, we can follow a top-down approach to extract potential host mechanisms and additional drugs targeting the proteins participating in these mechanisms. As an example, we identify 69 drugs currently in clinical trials for COVID-19 and group them based on their Anatomical Therapeutic Chemical classification (Supplementary Table 5)^22^. We focus on drugs from the immunostimulants class (L03) and their target proteins as starting seeds. We further select the interactors of the immune-related viral proteins ORF9B, ORF6, ORF3B, and ORF3A^10^ as end-point seeds. By applying the multi-Steiner tree algorithm, we discover pathways of interacting proteins that connect the selected drugs (and their target proteins) with the selected viral proteins. Among these connector proteins, we find five genes associated with cytokine signaling in the immune system according to Reactome Pathways (*CSF2*, NRG1, *NUP188*, *PTPN18*, *SOCS1*)^23^. Notably, *CSF2* is enriched in lung, pancreas, and immune cells (www.proteinatlas.org/ENSG00000164400-CSF2)^24^ and can be inhibited by KB002 (DB05194), which is an investigational drug and an engineered human monoclonal antibody treatment for inflammatory and autoimmune processes^25^. In summary, with CoVex, we found a new drug target that may play a key role in the host immune response during viral infection. We also identified a new drug candidate for COVID-19, as it targets the proteins involved in the pathogenic mechanisms triggered by ORF3A, ORF3B, ORF6, and ORF9B viral proteins.

*Scenario d*: Starting from a hypothesis-driven mixed selection of viral and host proteins, as well as drugs, we seek to utilize PPIs to identify a full mechanism or pathway and to suggest additional drug candidates. As an application case, we follow-up on a recently published hypothesis by Liu and Abrahams concerning the putative interference of SARS-CoV-2 with the formation of hemoglobin in erythrocytes^26,27^. Essentially, the virus is believed to interfere with heme formation causing symptoms of hypoxia. Liu and Abrahams hypothesize that this would also explain why Chloroquine and Favipiravir are effective drugs, as they may prevent the viral proteins from competing with iron for the porphyrin in hemoglobin (NSP1-16, ORF3a, ORF10, and ORF8 targeted by Chloroquine as well as ORF7a targeted by Favipiravir)^26,27^. Based on this hypothesis (discussed in more detail in the Supplementary Notes), we investigate the pathways connecting these viral proteins with the two effective drugs Chloroquine and Favipiravir. To this end, we select two known heme binding host proteins as seeds: cytochrome b5 reductase, which interacts with the viral protein NSP7, and the viral ORF3a, which binds to heme oxygenase 1. Using KeyPathwayMiner for drug target discovery followed by closeness centrality for drug discovery, we identify methylene blue in addition to Chloroquine and Deferoxamine, which are both in COVID-19 clinical trials^28,29^. Notably, methylene blue is approved by the Food and Drug Administration for the treatment of methemoglobinemia, which fits the investigated hypothesis (reduced oxygen-carrying capacity). Also, Deferoxamine is widely used therapeutically as a chelator of ferric ions in disorders of iron overload^30^. However, note that the available scientific evidence for a methemoglobinemia or ferric ion imbalance caused by SARS-CoV-2 is very limited (see Supplementary Notes) and that we use this hypothesis solely to illustrate the potential of CoVex’ network medicine investigation and hypothesis testing capabilities.

## Discussion

COVID-19 is a threat to our health and our social life, as well as to our healthcare and economic systems around the globe. Since the development of safe and effective vaccines is a time-consuming process, the only alternative to mitigate the damage by the SARS-CoV-2 pandemic is to quickly identify agents for the treatment and control of COVID-19 symptoms. Much attention in biomedical and clinical research is, thus, given to the task of identifying therapeutically exploitable drugs. A particular interest lies in drug repurposing, since already approved drugs can go through shortened clinical trials within months rather than years. While a number of promising drug repurposing candidates are currently being tested, the discovery of such candidates is still unstandardized and mostly unstructured. Systems and network medicine offer alternative approaches, where the process of drug target discovery is driven by computational data mining methods utilizing molecular interaction networks. As recently demonstrated by Gysi et al.^11^ for SARS-CoV-2, this data-driven process can produce a list of promising drug candidates targeting host proteins in close proximity and mechanistically related to virus-interacting proteins^11^. Here, we seek to make this network medicine approach widely available to the community.

With CoVex, we present an interactive and user-friendly web platform that integrates published data of SARS-CoV-1 as well as recent data about virus–host interactions in SARS-CoV-2^10^ with the human interactome and several drug–target interaction databases. CoVex allows users to mine the integrated virus–host–drug interactome for putative drug targets and drug repurposing candidates with only a few mouse clicks. Through features such as interactive seed protein selection, filtering, and upload of own lists of proteins or drugs of interest, CoVex covers diverse application scenarios ranging from data-driven, hypothesis-free drug target discovery to expert-guided analyses with a clear underlying hypothesis about virus biology. To address the diversity of research questions adequately, CoVex implements several state-of-the-art graph analysis methods. These were specifically tailored to be employed in a network medicine context and include a weighted version of TrustRank as well as a multi-Steiner tree method (Supplementary Material).

While CoVex is a powerful tool for SARS-CoV-1 and -2 research, results uncovered with our platform have to be considered with caution. We stress that CoVex can only suggest putative drug candidates for further investigation and that those candidates are not guaranteed to have an antiviral effect. While the suggested drugs target proteins involved in a putatively important mechanism for the virus, the actual effect of the drug has to be verified through follow-up investigations. The inhibition of a cofactor that prevents the virus from manipulating host proteins, for example, could even have a proviral effect. After validating the target for the suggested drug through appropriate genetic or chemical approaches, the drug candidate, hence, still needs to be properly vetted by clinical experts and tested following established procedures and clinical trials. Current data about virus–host interactions in SARS-CoV-2 is still preliminary and incomplete. For instance, important proteins such as the ACE2 receptor, a known entrypoint for the virus^18^, is missing in the SARS-CoV-2 dataset by Gordon et al.^10^. Moreover, we included only drugs that are reported in databases about clinical trials or in the literature if they have a valid entry in DrugBank, possibly excluding some of the drugs currently being investigated. Further, we do not differentiate between different sources of drug–target interactions. The strength of experimental evidence may vary depending on the experimental assay that was used or the type of annotation from the source database, for example, clinical and variant annotations from PharmGKB, which can be interpreted as indirect drug–protein associations. It should also be noted that we do not list drugs that target viral proteins directly, as the goal of CoVex is to unravel novel drug targets further downstream in the human interactome.

We acknowledge that the choice of algorithm and its associated parameters is nontrivial, forcing users to engage in time-consuming explorative analysis. To make this easier, we allow users to queue multiple tasks, which are executed in parallel. As our experience with this platform grows, we also plan to develop guidelines that allow users to choose an appropriate method for a particular research question. We further plan to integrate new data about virus–host interactions and ongoing clinical trials in corona viruses as it becomes available.

In summary, we have presented CoVex, a web-based platform for the interactive exploration and network-based analysis of virus–host interactions, aimed towards drug repurposing for the treatment of COVID-19. CoVex can be easily updated to accommodate the fast-paced data generation in the battle against the global pandemic. CoVex is expected to speed up the discovery of potential therapeutics for COVID-19. For the future, we also plan to extend the CoVex network medicine platform to other viruses in which new drug targets and drug repurposing candidates are urgently sought, including MERS, Zika, influenza, and dengue. We will also add features for the integration of additional molecular data, such as gene expression. Until then users can work with the “add custom protein” functionality of CoVex, allowing them to utilize and filter by any set of genes, including those derived by gene expression pattern analyses.

## Methods

### Data integration

We integrated virus–host interaction data from several sources. We obtained SARS-CoV-2 AP-MS data reported by Gordon et al.^10^, containing 332 high-confidence virus–host interactions for 27 SARS-CoV-2 proteins^10^, as well as SARS-CoV-1 interactions from VirHostNet^4^ (24 interactions), and Pfefferle et al.^31^ (113 interactions existing in our interactome). Human PPIs were obtained from the integrated interactions database^32^ filtered based on experimental validation. The resulting interactome consists of 17,666 proteins connected via 329,215 interactions. Drug–target associations were obtained from ChEMBL (2020-03)^33^, DrugBank (v. 5.1.5)^25^, DrugCentral (2018-08-26)^34^, Target Therapeutic Database (2019-07-14)^35^, Guide To Pharmacology (2020-01; only approved drugs)^36^, PharmGKB (downloaded 2020-04)^37^, and BindingDB (2019-08-12)^38^. Where applicable, we considered drugs that have binding affinity values (EC50, IC50, *K*_d_, and *K*_i_) <10  μM^39,40^. Only drugs that were mappable to DrugBank IDs and targeting host proteins were included in the network. Drugs currently undergoing clinical trials and mappable to DrugBank IDs (as of April 4, 2020) for the treatment of COVID-19 were collected from ClinicalTrials.gov (www.ClinicalTrials.gov)^41^, the EU Clinical Trials Register (www.clinicaltrialsregister.eu), and the International Clinical Trials Registry Platform (www.who.int/ictrp/). In total, we have 6861 drugs (67 in clinical trials) and 52,860 drug–target associations integrated in our network. We further downloaded per-tissue median gene expression levels from the GTEx data portal (Release V8, dbGaP Accession phs000424.v8.p2, downloaded 2020-05-30) to allow for tissue-specific filtering and visualization of gene expression values. Note that we rely on integrating published data and, thus, on their corresponding quality.

### Systems medicine algorithms for drug repurposing prediction

The general idea of CoVex is to provide researchers and clinicians with a tool to visually explore druggable molecular mechanisms that drive the interactions between virus and host. To this end, the integrated virus–human–drug interactions form molecular networks that are modeled as graphs with nodes as proteins or drugs, and edges referring to interactions between them. The goal of CoVex is to explore this network while allowing for the exploitation of expert knowledge. Starting with a selected set of (usually) hypothesis-driven seeds (virus proteins, human proteins, or drugs), the goal is to first identify subnetworks connecting these seeds and, subsequently, to identify drug repurposing candidates associated with these mechanisms. A vast number of methods have been reported in the literature for identifying subnetworks^42^. In CoVex, we have integrated several algorithms (including a dedicated multi-Steiner tree algorithm) with different underlying paradigms to provide specific exploration options to various particular medical, therapeutic, and research questions and hypotheses. CoVex, thus, allows users to choose among the following approaches in the “advanced analysis” procedures.

Degree centrality is the simplest conceivable centrality measure and ranks proteins or drugs interacting with the seeds by their node degree, that is, the number of interactions. Thus, this algorithm yields subnetworks in which seed-connected proteins and/or drugs are preferentially selected if they interact with many other proteins in the network. The only user-selected parameter is the result size, that is, how many of the top-ranked proteins or drugs are included. Notably, centrality measures in CoVex can be used for detecting drug targets and for identifying promising drugs.

Closeness centrality is a node centrality measure that ranks the nodes in a network based on the lengths of their shortest paths to all other nodes in the network. The rationale behind this algorithm is to preferentially select proteins and/or drugs that are a short distance from all other proteins in the network and are thus of central importance. In CoVex, we use a modified version suggested by Kacprowski et al.^43^, where only the shortest paths to a set of selected seed nodes are considered. The only algorithm-specific, user-selected parameter is the result size.

Betweenness centrality is another node centrality measure that ranks the nodes in a network based on how many shortest paths pass through them. In CoVex, we use a modified version suggested by Kacprowski et al.^43^, which only considers shortest paths between pairs of seed nodes. Hence, nodes receive a high score if they are on many shortest paths between the seeds. Since drugs are not contained in any shortest paths in our integrated interactome (see Fig. 1), betweenness centrality can be used only to find drug targets. The only algorithm-specific, user-selected parameter is the result size.

Guney et al.^44^ introduced the network proximity between a drug and a set of seed nodes as the average minimum distance from the drug’s targets to all of the seeds. The algorithm computes empirical *z*-scores by comparing the obtained proximity score to a background distribution obtained by randomly sampling sets of seed nodes and drug targets. In CoVex, network proximity can be employed to find drugs, given a set of host proteins of interest. The user can specify the result size, as well as the number of randomly sampled instances used for computing the background distribution.

TrustRank is conceptually similar to closeness centrality but additionally considers the importance of the seed nodes themselves. In other words, TrustRank ranks nodes in a network based on how well they are connected to a (trusted) set of seed nodes^45^. It is a variant of Google’s PageRank algorithm, where “trust” is iteratively propagated from seed nodes to neighboring nodes using the network structure. The node centralities are initialized by assigning uniform probabilities to all seeds and zero probabilities to all non-seed nodes. In CoVex, the TrustRank algorithm can be run starting from a user-defined set of (trusted) seed proteins to obtain a ranked list of proteins in the PPI network that could be prioritized as putative drug targets. Similarly, TrustRank can be executed on the joint protein–drug interactome to identify drug repurposing candidates. User-selected parameters include the result size and the damping factor (range 0–1), which controls how fast “trust” is propagated through the network. A small damping factor results in a conservative behavior of the algorithm (nodes close to the seeds receive much higher scores than distant ones), while a large damping factor makes its behavior more explorative.

The Steiner tree problem is a classical combinatorial optimization problem. It aims at finding a subgraph of minimum cost connecting a given set of seed nodes. For CoVex, we have developed a weighted multi-Steiner tree method that computes approximate weighted multiple Steiner trees and connects them to one subnetwork. The user can select the set of proteins of interest and extract subnetwork(s) that connect the selected seed proteins as candidate mechanism(s) involved in COVID-19 progression. In this mechanistic subnetwork(s), we can then extract essential proteins and, thus, the most promising drug targets and repurposable drugs for COVID-19. User-selected parameters include the number of Steiner trees to be merged as well as the tolerance towards accepting more expensive subnetworks (for speeding up the approximation algorithm; for details see Supplementary Methods).

KeyPathwayMiner is a network enrichment tool that identifies condition-specific subnetworks (key pathways)^46^. In CoVex, we utilize the KeyPathwayMiner web service to extract a maximally connected subnetwork starting from a user-defined set of proteins of interest (seeds). The only user-selected parameter is *K*, which represents the number of permitted exception nodes, that is, proteins that were not part of the seed proteins but serve to connect them. Since these proteins act as bridges, these may represent key proteins participating in the dysregulated subnetwork even though they are not directly targeted by the virus and are therefore promising candidates for intervention. In its current implementation, exception nodes will only be added if they indeed possess a bridging characteristic and will not be shown otherwise.

Irrespective of the network analysis method used, the extracted solutions have a higher intrinsic probability to contain high-degree nodes (hubs), that is, proteins that have a large number of interactions. While these proteins are key players in the human interactome, they are not necessarily suitable drug targets as perturbing them might lead to severe unintended side effects. Since it is more likely that hub proteins are involved in several mechanisms and are not specific to the mechanism of the disease under study, users can also perform the analysis with the hub penalty, which can potentially favor more specific mechanisms related to COVID-19. To mitigate this bias, users can either select an upper bound to filter out high-degree nodes or, alternatively, penalize high-degree nodes by incorporating the degree of neighboring nodes as edge weights in the optimization. For the latter, values between 0 and 1 can be selected, where higher values correspond to a higher penalty. Both options are available in advanced analyses for all methods except for degree centrality, because its rationale is to identify hubs, and KeyPathwayMiner, which conceptually does not allow for weighted subnetwork extraction.

All network algorithms except multi-Steiner tree and KeyPathwayMiner yield scores for the nodes contained in the returned subnetwork. In the case of degree centrality, closeness centrality, betweenness centrality, and TrustRank, these scores correspond to, respectively, the number of direct interactions with the seeds, the inverse of the mean distance to the seeds, the fraction of shortest paths between the seeds passing through the node, and the “trust” on the node at termination. In all four cases, high scores indicate that the nodes are central with respect to the seeds, but the scores do not carry any intrinsic statistical semantics. In CoVex, we hence display normalized scores for degree centrality, closeness centrality, betweenness centrality, and TrustRank, which we compute by dividing by the obtained maximum. In contrast to that, network proximity yields empirical *z*-scores, which are smaller the more promising the drugs are for the selected set of seed proteins. Since these *z*-scores directly translate into empirical *p* values, we do not normalize them.

### Implementation

CoVex consists of five components: (i) Data are stored in a PostgreSQL database (v. 12.2). (ii) The backend is implemented using the Django web framework (v. 3.0.5) with Python (v. 3.6) and the Django REST framework (v. 3.11.0) to build the web API. (iii) The network algorithms (except KeyPathwayMiner) are implemented with graph-tool (v. 2.3.1)^47^. (iv) Background task processing is implemented using Redis Queue (RQ, v. 1.3.0) and the in-memory database Redis (v. 3.4.1). Django enqueues the jobs and RQ processes them in the background while Redis functions as a broker between Django and RQ. (v) The frontend is implemented in Angular (v. 9.0.2) and utilizes the JavaScript libraries vis-data (v. 6.5.1) and vis-network (v. 7.4.2) for network visualization.

### Reporting summary

Further information on research design is available in the Nature Research Reporting Summary linked to this article.

## Supplementary information

Supplementary Information

Reporting Summary

## Data Availability

The authors declare that all data supporting the findings of this study are available publicly and their integration is described accordingly within the paper and its supplementary information files. Human protein–protein interactions were obtained from the Integrated Interactions Database (http://iid.ophid.utoronto.ca/). Virus–host interactions were downloaded from VirHostNet (http://virhostnet.prabi.fr/). Drug–target associations were integrated from the following databases: ChEMBL (https://www.ebi.ac.uk/chembl/), DrugBank (https://www.drugbank.ca/), DrugCentral (http://drugcentral.org/), Target Therapeutic Database (http://bidd.nus.edu.sg/group/cjttd/), Guide To Pharmacology (https://www.guidetopharmacology.org/), PharmGKB (https://www.pharmgkb.org/), and BindingDB (https://www.bindingdb.org/bind/index.jsp). Drugs undergoing clinical trials for COVID-19 were collected from ClinicalTrials.gov (https://clinicaltrials.gov/), the EU Clinical Trials Register (https://www.clinicaltrialsregister.eu/), and the International Clinical Trials Registry Platform (https://www.who.int/ictrp/en/). Tissue-specific gene expression levels were obtained from the GTEx data portal (https://www.gtexportal.org/home/, dbGaP Accession phs000424.v8.p2).

## Code availability

CoVex is a public online platform software running on a web server. The CoVex code is available from the corresponding author upon reasonable request. The online tool is available at https://exbio.wzw.tum.de/covex/.
