# Supplementary material for: Exploring the SARS-CoV-2 virus-host-drug interactome for drug repurposing
Source: Nat Commun. 2020 Jul 14;11:3518. doi: 10.1038/s41467-020-17189-2 (PMC7360763; doi:10.1038/s41467-020-17189-2)
Supplement: Supplementary file 1 — Supplementary Information [file 41467_2020_17189_MOESM1_ESM.pdf]

Supplementary Information

Exploring the SARS-CoV-2 virus-host-drug interactome for drug repurposing

Sadegh *et al.*

Supplementary Methods

Here, we present the new algorithmic techniques we developed to analyze the virus-host-drug interactome by starting from a set of seed nodes, avoiding hubs. Moreover, we give additional, detailed information about the application cases described in our CoVex paper.

New models and algorithms for seeded, weighted network analysis with hub-penalties

Multi-Steiner trees: The Steiner tree problem is a classical combinatorial optimization problem. Given an undirected graph $G=(V,E)$ with non-negative edge costs c and a set of seed nodes $S \subseteq V$, it asks to compute a minimum-cost Steiner tree for S, where a Steiner tree for S is a tree in G that contains all nodes $v \in S$. Exactly solving this problem is NP-hard, but several efficient approximation algorithms exist.

In CoVex, we compute (several) Steiner trees to connect selected host or viral proteins and extract a potentially druggable mechanism. For this, we developed a customized version of the 2-approximation algorithm by Kou et al. [1,2]. There are two main differences: First, we do not only compute one but return the union of K Steiner trees, where K is a parameter that can be set by the user. Computing several Steiner trees increases the stability of the extracted mechanism. This is important, because the networks used in CoVex are very dense, which implies that solutions to the Steiner tree problem are usually non-unique. Which solution is returned is, hence, a matter of chance, if only one Steiner tree is computed. Returning the union of K Steiner trees mitigates this problem. Second, we employ special parameterized edge costs c_{λ} with a hub-penalty $\lambda \in [0,1]$ that can be specified by the user (detailed below). If λ is set to a value close to 1, the returned Steiner trees avoid hub-nodes. This is important, because targeting proteins which are hub-nodes in the human protein-protein interaction (PPI) network potentially results in many undesired side effects.

The multi-Steiner tree algorithm employed in CoVex is implemented as follows: In a first step, we use the algorithm by Kou et al. to compute the first Steiner tree T. Moreover, we run a depth-first search to find all bridges in the graph, where a bridge is an edge whose deletion results in the graph being disconnected. Let L be the list of edges in T, C be the cost of T, and τ be a user defined tolerance that specifies by how much the costs of the subsequent trees may exceed C. Furthermore, let k be the number of already discovered trees (initialized to 1) and U be the set of returned nodes (initialized to the nodes contained in T). We iterate the following steps until $k=K$ or L is empty. Subsequently, we return the subgraph induced by U.

1. Pop edge e from L.
2. If e is a bridge, go to step 1.
3. Temporarily delete e from G.
4. Run the algorithm by Kou et al. to compute the next candidate tree T'.

5. If the cost of T' does not exceed $C \cdot \frac{100+\tau}{100}$, add the nodes of T' to U and increment k.
6. Remove all edges from L which are not contained in T'.
7. Reinsert e into G.

Weighted TrustRank: TrustRank is a variant of Google's pagerank algorithm, where "trust" is iteratively propagated through the network starting from an initial set of (trusted) seed nodes [3]. At termination, each node in the network receives a score which is high if the node is easily reachable from a subset of seed nodes, which themselves assume central positions in the overall topology of the network.

In CoVex, TrustRank can be used for two purposes. First, TrustRank can be used to rank drugs targeting a previously selected or computed set of host proteins. Second, it can be employed to discover potentially druggable host proteins which are relevant for a given set of viral or host seed proteins. Note that, unlike the result returned by the multi-Steiner algorithm, the top ranked nodes returned by TrustRank are not guaranteed to be connected. In practice, however, many of the returned nodes often form one large connected component. Thus, TrustRank allows to extract pathways in situations where it is not clear *a priori* that all seed nodes are involved in one mechanism.

In CoVex, we use a customized version of the TrustRank algorithm which allows the user to avoid hubs, if required. This is achieved by defining parameterized edge diameters $d_{\lambda}(e) = 1/c_{\lambda}(e)$ (the definition of c_{λ} is given below in section "Parameterized edge costs with hub-penalty"). If the user sets the hub-penalty λ to a value close to 1, the diameters of the edges which are incident with hub-nodes are small. Therefore, "trust" flows less easily to the hubs, which implies that they obtain a lower score. Further parameters are the result size, which specifies how many top ranked nodes should be displayed in the result, and the damping factor $d \in [0, 1]$. The damping factor d controls how easily "trust" can flow to nodes which are far away from the seeds. The larger d, the higher scores distant nodes receive.

Weighted, seeded closeness centrality: Closeness centrality is a simple centrality measure which ranks the nodes in a network based on the average distance of the shortest paths to all other nodes. Kacprowski *et al.* suggested a version of this centrality measure where only the distances to a selected set of seed nodes are taken into consideration [4].

In CoVex, we use a modified version of this approach which uses the parameterized edge costs c_{λ} instead of uniform costs. This customized version of seeded closeness centrality hence assigns lower scores to hubs, if requested by the user. Like TrustRank, closeness centrality can be used both for extracting drug targets and for ranking drugs.

Parameterized edge costs with hub-penalty: For each edge $(u, v) \in E$ in the network G, the parameterized costs employed by the three algorithms presented above are defined as $c_{\lambda}(u, v) = (1 - \lambda) \cdot avdeg(G) + \lambda \cdot \frac{deg(u) + deg(v)}{2}$, where $deg(u)$ and $deg(v)$ are the degrees (i.e.,

number of links) of the nodes u and v, $avdeg(G)$ is the mean degree of all nodes contained in G, and $\lambda \in [0,1]$ is a hub-penalty, which can be specified by the user. Note that for $\lambda = 0$, all edge costs equal $avdeg(G)$ (no hub-penalty), while for $\lambda = 1$, the cost of each edge is a direct function of the degrees of its incident nodes (maximal hub-penalty). By setting λ to a value between 0 and 1, the user can balance between these two extremes.

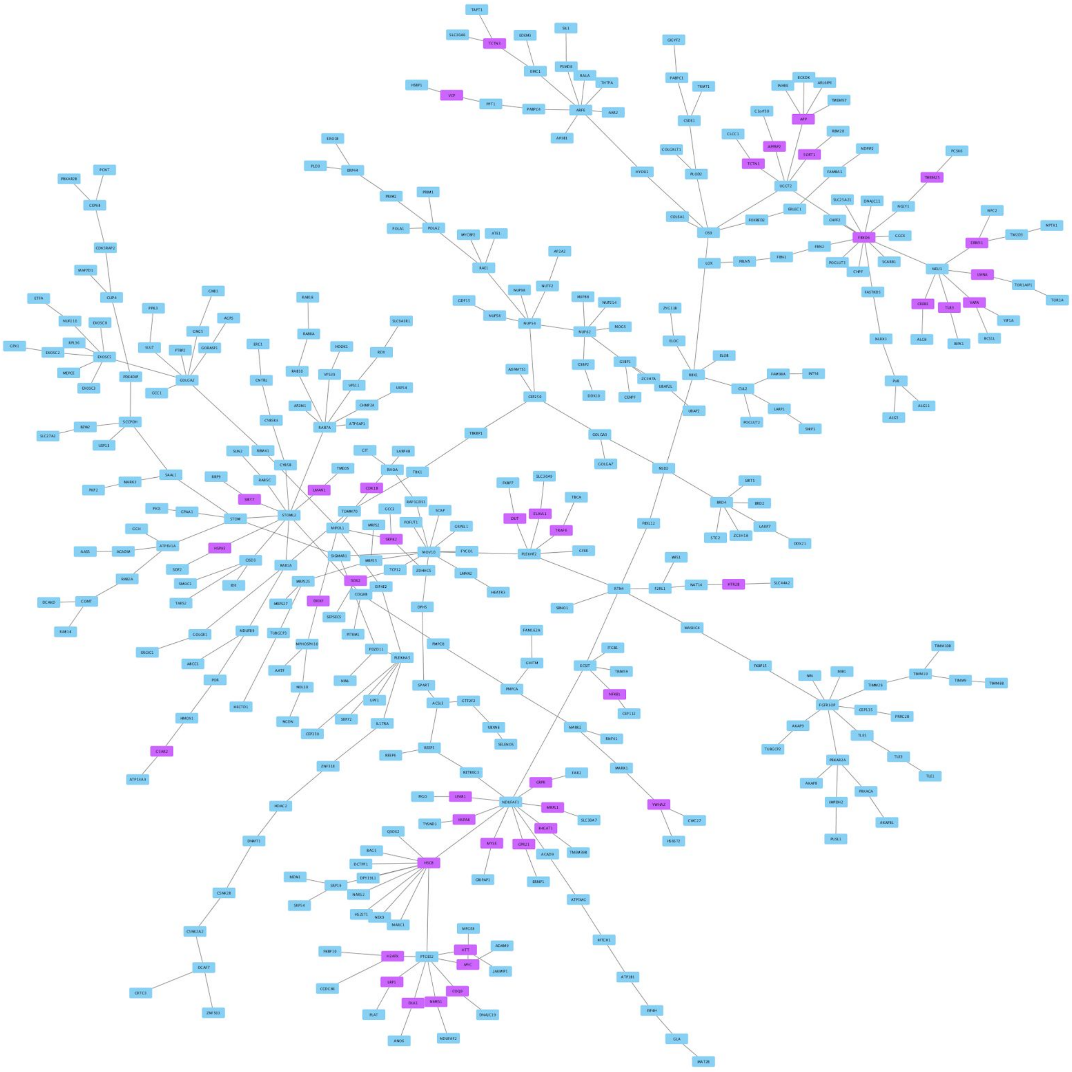

Supplementary Figure 1 - The multi-Steiner tree connecting all host proteins interacting with the virus (blue nodes are seeds) visualized in Cytoscape [5] using the GraphML export feature of CoVex. The purple nodes are the connectors building a tree structured subnetwork of the human interactome.

Why the host protein interactions matter for drug repurposing

247 out of 332 host proteins interacting with the virus (interactors) reported in Gordon *et al.* [6] for SARS-CoV-2 form a large connected component, indicating that most of the direct targets of the

virus are in a close proximity in the human interactome. 12 drugs currently in clinical trials (Dexamethasone, Colchicine, Pravastatin, Ribavirin, Ruxolitinib, Bromhexine, Oseltamivir, Noscapine, Ascorbic acid, Tofacitinib, Artenimol, Suramin) target the virus interactors directly. Supplementary Figure 1 shows a Steiner tree of minimum cost connecting all the interactors. It consists of 44 connector proteins in addition to 332 interactors. The Steiner tree enables us to find new drug target candidates. Importantly, six of the 69 drugs currently in clinical trials (Supplementary Table 5) target exclusively the connector proteins revealed by our analysis, namely Glycyrrhizic acid, Synthetic Conjugated Estrogens B, Leflunomide, Chloroquine, Deferoxamine, and Thalidomide.

Supplementary Notes

Application scenario a - Starting from a selection of viral proteins, we seek to use the human interactome to identify biological mechanisms or pathways utilized by the virus during infection. As an example, we are interested in the viral proteins E, M and Spike, which constitute the external structure of the virus and thus participate in the entry into host cells [7,8].

First, we select all host proteins interacting with the viral proteins E, M and Spike from the SARS-CoV-2 dataset. We then use the multi-Steiner Tree algorithm with the parameters shown in Supplementary Table 1 to uncover the biological pathway involved. The resulting network allows the identification of 26 new potential drug targets, including the Bradykinin receptor B1 (BDKRB1).

Next, we use closeness centrality with the parameters shown in Supplementary Table 1 to find drugs affecting this pathway. We identify a total of 30 approved and 10 non-approved drugs (Supplementary Figure 2). Notably, we find 6 relevant drugs that target BDKRB1: Ramipril, Captopril, Perindopril and Enalaprilat (approved), which belong to the Angiotensin Converting Enzyme (ACE) inhibitor class [9]. Icatibant is an antagonist of the Bradykinin receptor B2 [10] and bradykinin is a non-approved drug which is degraded by the ACE [11].

Finally, to understand the relationship between BDKRB1 and Angiotensin Converting Enzyme 2 (ACE2) as well as Transmembrane protease serine 2 (TMPRSS2), two proteins known to be involved during virus entry [12], we use the “custom proteins” option available in CoVex and utilize the multi-Steiner tree algorithm with the same parameters as in Supplementary Table 1. We find that the Kininogen 1 (KNG1) and Angiotensin (AGT) proteins connect BDKRB1 with ACE2 (Supplementary Figure 3). These 4 proteins are functionally related through the Renin-Angiotensin System, which is targeted by ACE inhibitors (https://www.wikipathways.org/instance/WP554).

In summary, CoVex identifies the protein BDKRB1, which participates in the pathway affected by SARS-CoV-2 and can be targeted by several ACE inhibitors, which are widely used in clinical trials to treat COVID-19. It should be noted that the ACE2 protein is not present in the set of seeds used to start the analysis; however, CoVex is capable of identifying the pathway and new

protein targets functionally related to ACE2, which can be targeted by ACE inhibitors as well. In this case, CoVex allows the identification of the mechanism behind the drugs currently considered for treating currently considered for treating COVID-19.

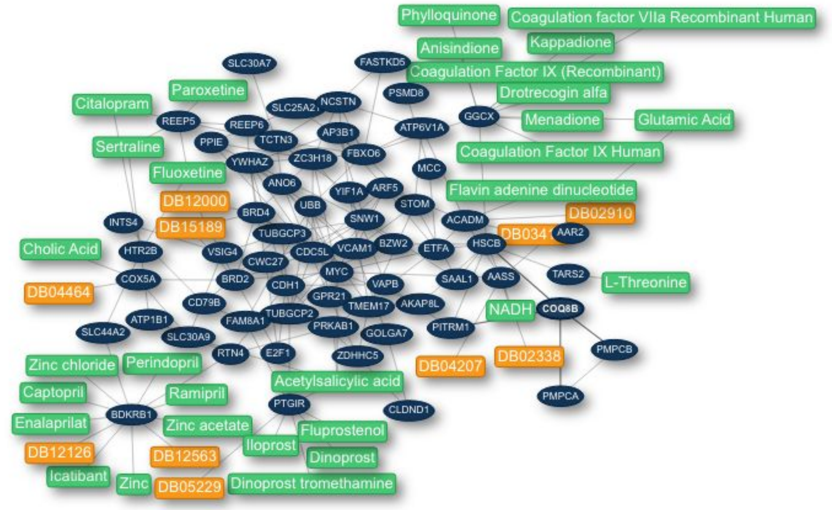

Supplementary Figure 2 - Network obtained from interactors of baits E, M and Spike with multi-Steiner tree followed by closeness centrality. Blue nodes are protein targets, green nodes are approved drugs and orange nodes are non-approved drugs. Lines represent the interactions between the proteins and drugs. ACE inhibitor drugs are identified, such as Ramipril, Captopril, Perindopril and Enalaprilat targeting the BDKRB1 protein, which are currently being evaluated in clinical trials in COVID-19 patients. Note that we also included this Figure in the main document (Figure 4) to showcase how CoVex results look like but kept it here as well for increased readability.

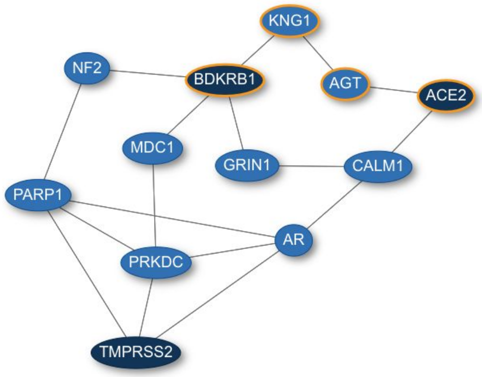

Supplementary Figure 3 - The subnetwork obtained with the multi-Steiner tree algorithm shows the connections between ACE2, TMPRSS2 and BDKRB1. Dark blue nodes represent the seed proteins and light blue nodes represent the connector proteins identified.

Supplementary Table 1 - Algorithms and parameters used in application scenario a

	Algorithm	
	multi-Steiner tree	Closeness centrality
Number of trees	10	N.A.
Tolerance	0	N.A.
Result size	N.A.	40
Maximum degree	DISABLED	DISABLED
Hub penalty	0	1
Include non-seed viral proteins	FALSE	N.A.
Include indirect drugs	N.A.	FALSE
Include non-approved drugs	N.A.	TRUE

Application scenario b - Candidate drugs for the treatment of COVID-19 can be identified starting from a user-defined set of seeds comprising differentially expressed genes (DEGs) and viral proteins. Such a custom list can be obtained from other datasets, such as experiments and/or literature. One possible strategy is to use proteins known to be associated with a specific biological process, such as SARS-CoV-2 proteins involved in viral pathogenesis and host proteins that participate in the corresponding host immune response to infection. This way, we use the host PPI network to connect the viral proteins to the DEGs and obtain a potential mechanism that can be targeted using repurposable drugs. To obtain a custom list of DEGs, raw counts from the gene expression data of SARS-CoV-2-infected lung epithelial A549 cells relative to mock-treated cells were obtained from Blanco-Melo *et al.* [13] (GEO accession GSE147507). Differential expression analysis using the edgeR (v.3.26.8) package (fold change ≥2, adjusted p-value <0.05) was performed to obtain a list of DEGs. To identify host cell pathways enriched in response to SARS-CoV-2 infection, KEGG enrichment was performed using the gseapy (v.0.9.15) package. Enriched KEGG terms included pathways that are known to be involved in immune response to pathogens, such as “Influenza A”, “Herpes simplex infection”, “Measles”, and “Hepatitis C”.

In this example, we use the CoVex platform to select viral proteins involved in innate immune response and apoptosis, namely, ORF7a and ORF3a, as indicated in Gordon *et al.* [6]. Next, using the “Custom proteins” option, we upload the Uniprot IDs of the DEGs that participate in the enriched pathway Herpes simplex infection, which is involved in response to infection with Herpes simplex virus (HSV), another viral pathogen. The enriched DEGs include *IFIH1*, *OAS1*, *STAT1*, *DDX58*, *OAS2*, *OAS3*, *IRF7*, *EIF2AK2*, *IFIT1*, and *IRF9*. We then use both the viral proteins and DEGs as seeds for the multi-Steiner tree algorithm to extract a subnetwork that is relevant to our pathway of interest, shown in Supplementary Figure 4. Next, we use closeness centrality on the resulting subnetwork to obtain drugs. The parameters used to run the multi-Steiner tree and closeness centrality algorithms can be found in Supplementary Table 2. Top-ranking drugs included Tofacitinib and Ruxolitinib, which are currently being assessed in clinical trials for the treatment of COVID-19 (Supplementary Figure 5). Tofacitinib and Ruxolitinib are both known to inhibit Janus kinase (Jak), which promote cytokine signaling [14,15]. Thus,

administration with these drugs can mitigate immune-mediated lung injury and prevent functional functional deterioration in COVID-19 patients caused by an over-amplified host immune response. As shown in Supplementary Figure 5, other drugs that target this subnetwork include Masitinib, Erlotinib, and Sorafenib, which could be further examined in downstream analyses. In a similar manner, users can provide a custom list of proteins to retrieve drugs that can target their mechanism of interest, followed by careful examination of the results.

Supplementary Table 2 - Algorithms and parameters used in application scenario b

	Algorithm	
	multi-Steiner tree	Closeness centrality
Number of trees	10	N.A.
Tolerance	0	N.A.
Result size	N.A.	20
Maximum degree	DISABLED	DISABLED
Hub penalty	0	0
Include non-seed viral proteins	TRUE	N.A.
Include indirect drugs	N.A.	FALSE
Include non-approved drugs	N.A.	FALSE

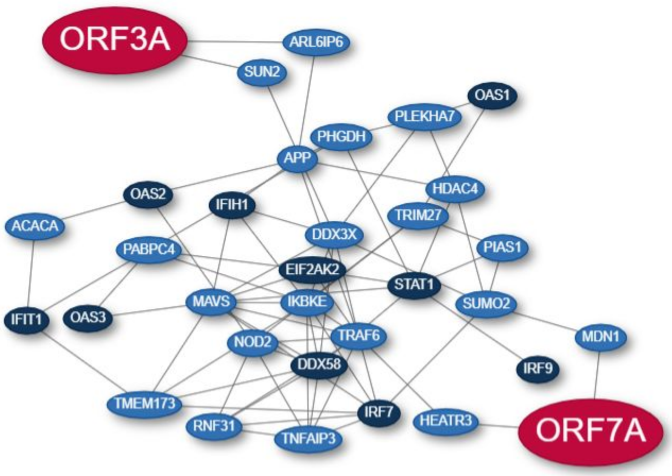

Supplementary Figure 4 - The subnetwork showing the connections between the viral proteins (ORF7A, ORF3A) and proteins involved in Herpes simplex infection. Dark blue nodes indicate proteins involved in Herpes simplex infection, while bright blue nodes indicate the connector nodes found by the multi-Steiner tree algorithm. The resulting subnetwork comprises potential drug targets to suppress the host immune response.

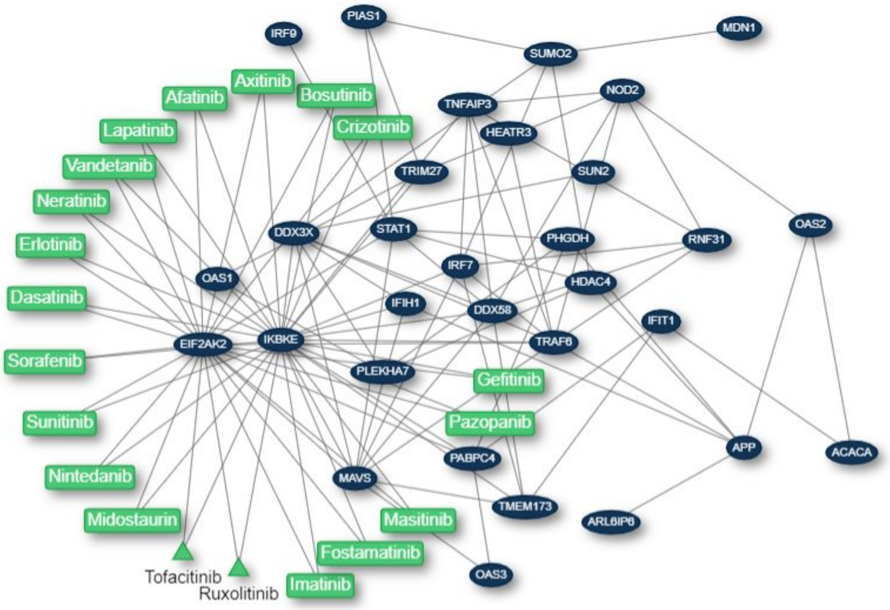

Supplementary Figure 5 - Closeness centrality recovered drugs, such as Tofacitinib and Ruxolitinib, which are currently being evaluated in clinical trials in COVID-19 patients.

Application scenario c - Another approach to find candidate drugs to combat COVID-19 is to connect the targets of promising drugs which are already in clinical trials to the viral proteins. We can identify the candidate mechanisms by extracting the sub-network(s) connecting these two ends with a minimum number of intermediate connector proteins. This step can be done by using the multi-Steiner tree algorithm integrated in our CoVex platform. We can then seek for the drugs targeting the identified connector proteins utilizing the closeness centrality algorithm from the "Find Drugs" function.

As an example, we start with the drugs classified as immunostimulants (Sargramostim and Peginterferon alfa-2a)[16], and then use the multi-Steiner tree algorithm from the "Find Drug Targets" function to connect their targets (IL3RA, CSF2RB, SDC2, PRG2, IFNAR2, IFNAR1, and CSF2RA) to the host interactor genes of viral proteins ORF9B, ORF6, ORF3B, and ORF3A (ALG5, ARL6IP6, BAG5, CHMP2A, CLCC1, CSDE1, DCTPP1, DPH5, HEATR3, HMOX1, MARK1, MARK2, MARK3, MDN1, MTCH1, NUP98, PTBP2, RAE1, SLC9A3R1, STOML2, SUN2, TOMM70, TRIM59, VPS11, VPS39), see Supplementary Figure 6. Among the identified connector proteins, we find five genes (CSF2, NRG1, NUP188, PTPN18, SOCS1) associated with cytokine signaling in the immune system according to Reactome Pathways [17]. From this list, notably, CSF2 is enriched in lung and immune cells (www.proteinatlas.org/ENSG00000164400-CSF2) [18]. This gene can be inhibited by the investigational drug KB002 (DB05194) (Supplementary Figure 7), which is an engineered human monoclonal antibody-based treatment for inflammatory and autoimmune processes [19].

Supplementary Table 3 - Algorithms and parameters used in application scenario c

	Algorithm	
	multi-Steiner tree	Closeness centrality
Number of trees	10	N.A.
Tolerance	0	N.A.
Result size	N.A.	50
Maximum degree	DISABLED	DISABLED
Hub penalty	1	0
Include non-seed viral proteins	FALSE	N.A.
Include indirect drugs	N.A.	FALSE
Include non-approved drugs	N.A.	TRUE

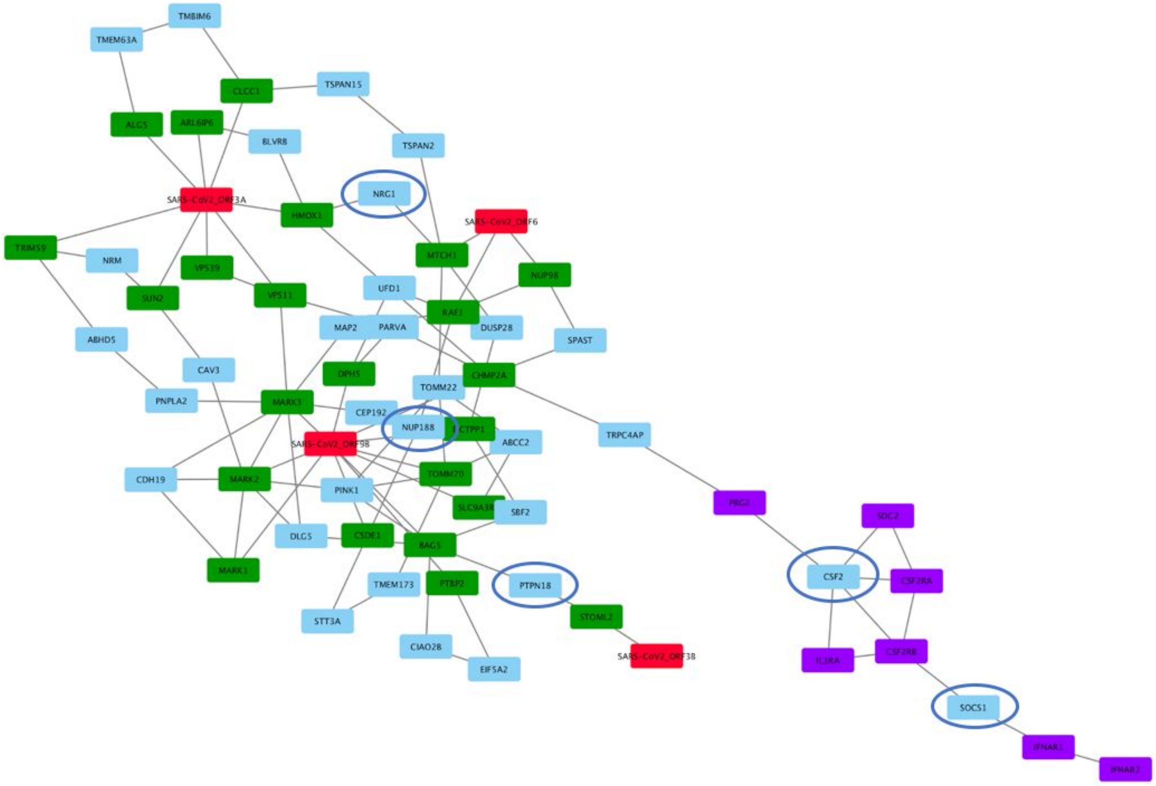

Supplementary Figure 6 - Cytoscape illustration of the multi-Steiner tree analysis performed with CoVex after using the export to GraphML feature, with targets of Sargramostim, Peginterferon beta-1a, and Peginterferon alfa-2a (purple) and the host interactor genes of viral proteins ORF9B, ORF6, ORF3B, and ORF3A (green) as seeds. Connector proteins (blue). Viral proteins (red). Connector genes associated with cytokine signaling in the immune system (circled in blue).

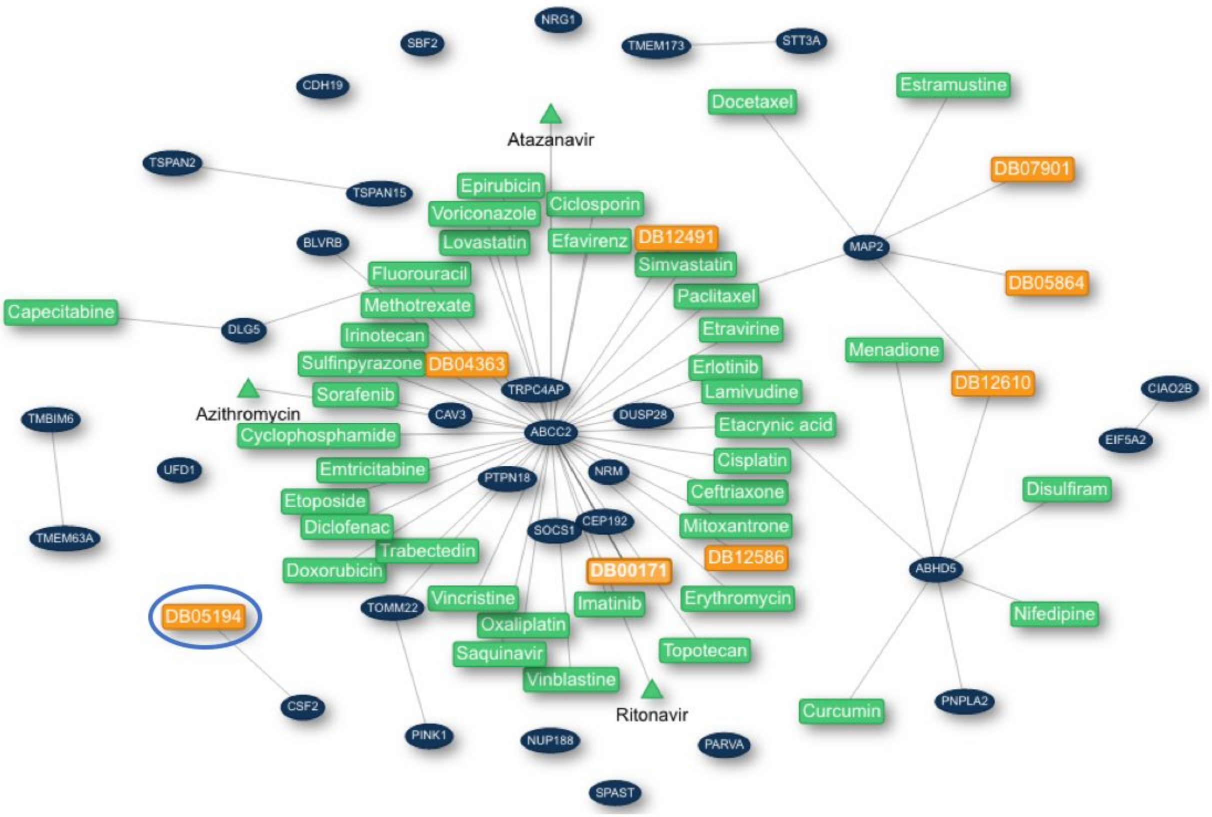

Supplementary Figure 7 - The closeness centrality algorithms ranks the drugs targeting the connector nodes from Supplementary Figure 6. The protein encoded by CSF2 is inhibited by drug KB002 (DB05194).

Application scenario d - Some hypotheses might require a hybrid seeding approach, where we are required to start from a hypothesis-driven mixed selection of viral and host proteins as well as drugs to explore protein-protein interactions to identify a mechanism and suggest additional drugs.

Here, we follow a recently published hypothesis concerning the interference of the SARS-CoV-2 with the formation of hemoglobin in erythrocytes [22,23]. Essentially, we follow the idea that the virus could interfere with porphyrin, which is a substrate, together with iron (Fe^{2+}) ions, during the synthesis of the heme prosthetic group in hemoglobin. The viral proteins thus hinder the interaction of iron with porphyrin, as the viral proteins are hypothesized to compete with iron for the porphyrin and thus to inhibit heme group synthesis, leading to hypoxia symptoms [24]. Liu and Abrahams suggest that this might explain why Chloroquine and Favipiravir are effective drugs, as they may prevent the virus from competing with iron for the porphyrin: Chloroquine by interfering with the viral proteins NSP1-16, ORF3a, and ORF10 which may bind the porphyrin to prevent heme synthesis, and also by inhibiting the binding of viral protein ORF8 to porphyrins

“to a certain extent” [24]. Favipiravir could prohibit ORF7a from binding to (free) porphyrin in addition to preventing the virus from entering the host cells [24].

Starting from this theory, we investigate the host interactome for potential drug repurposing candidates. In CoVex, we selected the viral proteins NSP1-16, ORF3a, ORF7a, ORF8 and ORF10 as seeds and expanded the network to all of their host partner proteins. Notably, we see that NSP7 may bind to Cytochrome b5 reductase (CYB5R), which converts methemoglobin to hemoglobin (oxygen-transporting Fe^{2+} hemoglobin to non-oxygen-transporting Fe^{3+} hemoglobin). Cytochrome b5 reductase is involved in the transfer of reducing equivalents from the physiological electron donor, NADH, via an FAD domain to the small molecules of cytochrome b5. It is also heavily involved in many oxidation and reduction reactions, such as the reduction of methemoglobin to hemoglobin [25–27]. In addition, we see that ORF3a binds to HMOX1 (Heme oxygenase 1), which might lead to interference with heme degradation. We continued with all 238 host interactors as new seeds and executed KeyPathwayMiner (Supplementary Table 4) to investigate the host interactome for proteins that connect the selected virus-host interactions proteins. We discovered five new drug targets (the proteins APP, XPO1, TRIM25, HSCB, FBXO6) for which we extracted 20 drug candidates using the closeness centrality measure and only screening for currently approved drugs. In the resulting ranked list, we rediscovered Chloroquine as well as Deferoxamine, both of which are currently in clinical trial or discussed in the literature as candidate drugs for COVID-19 treatment. Note that Deferoxamine is widely used for the treatment of Thalassemia and as a chelator of ferric ion in disorders of iron overload [28]. In addition to these two drugs, we find Methylene blue, a drug that is approved by the FDA for the treatment of methemoglobinemia. Note that the evidence for a methemoglobinemia caused by SARS-CoV-2 is anecdotal (no reports on abnormal methemoglobin levels, iron metabolism markers, etc. exist) and we used this hypothesis to illustrate a potential hybrid level starting point for the network medicine investigation of a hypothesis using CoVex.

Supplementary Table 4 - Algorithms and parameters used in application scenario d

	Algorithm	
	KeyPathwayMiner	Closeness centrality
K	5	N.A.
Result Size	N.A.	20
Maximum Degree	N.A.	DISABLED
Hub Penalty	N.A.	0.5
Include indirect drugs	N.A.	FALSE
Include non-approved drugs	N.A.	FALSE

Supplementary Table 5 - All drugs in clinical trials targeting host proteins sorted by classification. We compiled a list of all drugs targeting human proteins currently registered in clinical governmental trial databases (see full paper).

Drug Classification	Class ID	Drug names
Immunostimulants	L03	Sargramostim,Peginterferon alfa-2a
Immunosuppressants	L04	Eculizumab,Thalidomide,Pirfenidone,Anakinra,Fingolimod,Tocilizumab,Sarilumab, Ixekizumab,Siltuximab,Leflunomide,Emapalumab,Tofacitinib,Adalimumab,Baricitinib
Antineoplastic agents	L01	Ruxolitinib,Bevacizumab
Gynecological Anti Infectives And Antiseptics	G01	Darunavir,Ascorbic acid,Sildenafil
Vitamins	A11	Calcitriol,Ascorbic acid
Ophthalmologicals	S01	Heparin,Dexamethasone,Acetylcysteine,Ascorbic acid,Methylprednisolone,Azithromycin
Antipsoriatics	D05	Calcitriol
Lipid Modifying Agents	C10	Pravastatin
Antiobesity Preparations;excl.Diet Products	A08	Phentermine
Antivirals For Systemic Use	J05	Atazanavir,Ribavirin,Oseltamivir,Cobicistat,Sofosbuvir,Ritonavir,Darunavir,Lopinavir
Urologicals	G04	Sildenafil
Antibacterials For Systemic Use	J01	Azithromycin
Other Respiratory System Products	R07	Nitric Oxide
Antiprotozoals	P01	Suramin,Hydroxychloroquine,Artenimol,Chloroquine
Psycholeptics	N05	Dexmedetomidine
Agents Acting On The Renin-angiotensin system	C09	Losartan
Allothertherapeuticproducts	V03	Oxygen,Deferoxamine,Acetylcysteine,Cobicistat
Other Gynecologicals	G02	Naproxen
Topical Products For Joint And Muscular Pain	M02	Naproxen
Anti Inflammatory And Antirheumatic Products	M01	Naproxen

Anesthetics	N01	Sevoflurane,Propofol
Anthelmintics	P02	Levamisole
Anti-acne preparations	D10	Dexamethasone,Methylprednisolone
Corticosteroids For Systemic Use	H02	Dexamethasone,Methylprednisolone
Corticosteroids;dermatological preparations	D07	Dexamethasone,Methylprednisolone
Antithromboticagents	B01	Heparin,Enoxaparin,Dipyridamole
Vasoprotectives	C05	Heparin,Dexamethasone
Nasal Preparations	R01	Ciclesonide,Dexamethasone
Ophthalmological And Cytological Preparations	S03	Dexamethasone
Otologicals	S02	Dexamethasone
Stomatological Preparations	A01	Dexamethasone
Antigout Preparations	M04	Colchicine
Drugs for obstructive airway diseases	R03	Ciclesonide
Coughandcoldpreparations	R05	Acetylcysteine,Bromhexine,Noscapine
Cardiac therapy	C01	Angiotensin II
Anti Hemorrhagic	B02	Camostat
Bile And Liver Therapy	A05	Glycyrrhizic acid

Supplementary References

1. Agrawal, A., Klein, P. & Ravi, R. When Trees Collide: An Approximation Algorithm for the Generalized Steiner Problem on Networks. *SIAM Journal on Computing* vol. 24 440–456 (1995).
2. Kou, L., Markowsky, G. & Berman, L. A fast algorithm for Steiner trees. *Acta Informatica* vol. 15 141–145 (1981).
3. Gyongyi, Z., Garciamolina, H. & Pedersen, J. Combating Web Spam with TrustRank. *Proceedings 2004 VLDB Conference* 576–587 (2004) doi:10.1016/b978-012088469-8/50052-8.
4. Kacprowski, T., Doncheva, N. T. & Albrecht, M. NetworkPrioritizer: a versatile tool for network-based prioritization of candidate disease genes or other molecules. *Bioinformatics* **29**, 1471–1473 (2013).
5. Shannon, P. *et al.* Cytoscape: a software environment for integrated models of biomolecular interaction networks. *Genome Res.* **13**, 2498–2504 (2003).
6. Gordon, D. E. *et al.* A SARS-CoV-2-Human Protein-Protein Interaction Map Reveals Drug Targets and Potential Drug-Repurposing. *bioRxiv* 2020.03.22.002386 (2020) doi:10.1101/2020.03.22.002386.

7. de Haan, C. A. M. & Rottier, P. J. M. Molecular Interactions in the Assembly of Coronaviruses. in *Advances in Virus Research* vol. 64 165–230 (Academic Press, 2005).
8. Walls, A. C. *et al.* Structure, Function, and Antigenicity of the SARS-CoV-2 Spike Glycoprotein. *Cell* **181**, 281–292.e6 (2020).
9. Piepho, R. W. Overview of the angiotensin-converting-enzyme inhibitors. *Am. J. Health. Syst. Pharm.* **57 Suppl 1**, S3–7 (2000).
10. Icatibant. *Drugs in R & D* **5**, 343–348 (2004).
11. Kuoppala, A., Lindstedt, K. A., Saarinen, J., Kovanen, P. T. & Kokkonen, J. O. Inactivation of bradykinin by angiotensin-converting enzyme and by carboxypeptidase N in human plasma. *Am. J. Physiol. Heart Circ. Physiol.* **278**, H1069–74 (2000).
12. Hoffmann, M. *et al.* SARS-CoV-2 Cell Entry Depends on ACE2 and TMPRSS2 and Is Blocked by a Clinically Proven Protease Inhibitor. *Cell* (2020) doi:10.1016/j.cell.2020.02.052.
13. Blanco-Melo, D. *et al.* SARS-CoV-2 launches a unique transcriptional signature from in vitro, ex vivo, and in vivo systems. *bioRxiv* 2020.03.24.004655 (2020) doi:10.1101/2020.03.24.004655.
14. van Vollenhoven, R. *et al.* Evaluation of the Short-, Mid-, and Long-Term Effects of Tofacitinib on Lymphocytes in Patients With Rheumatoid Arthritis. *Arthritis Rheumatol* **71**, 685–695 (2019).
15. Elli, E. M., Baratè, C., Mendicino, F., Palandri, F. & Palumbo, G. A. Mechanisms Underlying the Anti-inflammatory and Immunosuppressive Activity of Ruxolitinib. *Front. Oncol.* **9**, 1186 (2019).
16. WHOCC. WHOCC - ATC/DDD Index. *WHOCC* https://www.whocc.no/atc_ddd_index/.
17. Cytokine Signaling in Immune system. *Reactome* https://reactome.org/content/detail/R-HSA-1280215.
18. Uhlen, M. *et al.* A pathology atlas of the human cancer transcriptome. *Science* **357**, (2017).
19. Wishart, D. S. *et al.* DrugBank 5.0: a major update to the DrugBank database for 2018. *Nucleic Acids Res.* **46**, D1074–D1082 (2018).
20. Hall, D. C., Jr & Ji, H.-F. A search for medications to treat COVID-19 via in silico molecular docking models of the SARS-CoV-2 spike glycoprotein and 3CL protease. *Travel Med. Infect. Dis.* 101646 (2020) doi:10.1016/j.tmaid.2020.101646.
21. Khan, S. A., Zia, K., Ashraf, S., Uddin, R. & Ul-Haq, Z. Identification of chymotrypsin-like protease inhibitors of SARS-CoV-2 via integrated computational approach. *J. Biomol. Struct. Dyn.* 1–10 (2020) doi:10.1080/07391102.2020.1751298.
22. Abrahams, L. Covid-19: acquired acute porphyria hypothesis. (2020) doi:10.31219/osf.io/4wkfy.
23. Wenzhong, L. & Hualan, L. COVID-19: Attacks the 1-Beta Chain of Hemoglobin and Captures the Porphyrin to Inhibit Human Heme Metabolism. (2020) doi:10.26434/chemrxiv.11938173.v7.
24. Liu, W. & Li, H. COVID-19: Attacks the 1-Beta Chain of Hemoglobin and Captures the Porphyrin to Inhibit Human Heme Metabolism.
25. Schomburg, D., Salzmann, M. & Stephan, D. Cytochrome-b5 reductase. *Enzyme Handbook 7* 225–231 (1994) doi:10.1007/978-3-642-78521-4_46.
26. Percy, M. J., McFerran, N. V. & Lappin, T. R. J. Disorders of oxidised haemoglobin. *Blood Rev.* **19**, 61–68 (2005).
27. Hay, D. & Weatherall, D. J. Disorders of the synthesis or function of haemoglobin. *Oxford Textbook of Medicine* 5426–5450 (2020) doi:10.1093/med/9780198746690.003.0537.
28. Lederman, H. M., Cohen, A., Lee, J. W., Freedman, M. H. & Gelfand, E. W. Deferoxamine:

a reversible S-phase inhibitor of human lymphocyte proliferation. *Blood* **64**, 748–753 (1984).
