# Supplementary material for: Exploring the SARS-CoV-2 virus-host-drug interactome for drug repurposing
Source: Nat Commun. 2020 Jul 14;11:3518. doi: 10.1038/s41467-020-17189-2 (PMC7360763; doi:10.1038/s41467-020-17189-2)
Supplement: Supplementary file 2 — Reporting Summary [file 41467_2020_17189_MOESM2_ESM.pdf]

nature research

Corresponding author(s): Jan Baumbach

Last updated by author(s): Jun 11, 2020

Reporting Summary

Nature Research wishes to improve the reproducibility of the work that we publish. This form provides structure for consistency and transparency in reporting. For further information on Nature Research policies, see our Editorial Policies and the Editorial Policy Checklist.

Statistics

For all statistical analyses, confirm that the following items are present in the figure legend, table legend, main text, or Methods section.

n/a	Confirmed	
x	☐	The exact sample size (n) for each experimental group/condition, given as a discrete number and unit of measurement
x	☐	A statement on whether measurements were taken from distinct samples or whether the same sample was measured repeatedly
x	☐	The statistical test(s) used AND whether they are one- or two-sided<br>*Only common tests should be described solely by name; describe more complex techniques in the Methods section.*
x	☐	A description of all covariates tested
x	☐	A description of any assumptions or corrections, such as tests of normality and adjustment for multiple comparisons
x	☐	A full description of the statistical parameters including central tendency (e.g. means) or other basic estimates (e.g. regression coefficient) AND variation (e.g. standard deviation) or associated estimates of uncertainty (e.g. confidence intervals)
x	☐	For null hypothesis testing, the test statistic (e.g. F, t, r) with confidence intervals, effect sizes, degrees of freedom and P value noted<br>*Give P values as exact values whenever suitable.*
x	☐	For Bayesian analysis, information on the choice of priors and Markov chain Monte Carlo settings
x	☐	For hierarchical and complex designs, identification of the appropriate level for tests and full reporting of outcomes
x	☐	Estimates of effect sizes (e.g. Cohen's d, Pearson's r), indicating how they were calculated

Our web collection on statistics for biologists contains articles on many of the points above.

Software and code

Policy information about availability of computer code

Data collection	The authors declare that all data supporting the findings of this study are available publicly and their integration is described accordingly within the paper and its supplementary information files.<br>The following packages and softwares are used for data storage, backend implementation and task processing: PostgreSQL database (v. 12.2), Django web framework (v. 3.0.5) with Python (v. 3.6), Django REST framework (v. 3.11.0), Redis Queue (RQ, v. 1.3.0), in-memory database Redis (v. 3.4.1)
Data analysis	CoVex is a public online platform software running on a web server. The CoVex code is available from the corresponding author upon reasonable request.<br>The following packages and softwares are used for implementation of the network algorithms, data and network visualization in the frontend: Graph-tool (v. 2.3.1), Angular (v. 9.0.2), JavaScript libraries vis-data (v. 6.5.1) and vis-network (v.7.4.2)

For manuscripts utilizing custom algorithms or software that are central to the research but not yet described in published literature, software must be made available to editors and reviewers. We strongly encourage code deposition in a community repository (e.g. GitHub). See the Nature Research guidelines for submitting code & software for further information.

Data

Policy information about availability of data

All manuscripts must include a data availability statement. This statement should provide the following information, where applicable:

- Accession codes, unique identifiers, or web links for publicly available datasets
- A list of figures that have associated raw data
- A description of any restrictions on data availability

The authors declare that all data supporting the findings of this study are available publicly and their integration is described accordingly within the paper and its

supplementary information files.
The following databases are used in this study: IID (http://iid.ophid.utoronto.ca/), VirHostNet (http://virhostnet.prabi.fr/), ChEMBL (https://www.ebi.ac.uk/chembl/), DrugBank (https://www.drugbank.ca/), DrugCentral (http://drugcentral.org/), Target Therapeutic Database (http://bidd.nus.edu.sg/group/cjttd/), Guide To Pharmacology (https://www.guidetopharmacology.org/), PharmGKB (https://www.pharmgkb.org/), BindingDB (https://www.bindingdb.org/bind/index.jsp). Drugs undergoing clinical trials for COVID-19 were collected from ClinicalTrials.gov (https://clinicaltrials.gov/), the EU Clinical Trials Register (https://www.clinicaltrialsregister.eu/) and the International Clinical Trials Registry Platform (https://www.who.int/ictrp/en/). Tissue-specific gene expression levels were obtained from the GTEx data portal Release V8, dbGaP Accession phs000424.v8.p2
(https://www.gtexportal.org/home/)

Field-specific reporting

Please select the one below that is the best fit for your research. If you are not sure, read the appropriate sections before making your selection.

- [x] Life sciences
- [ ] Behavioural & social sciences
- [ ] Ecological, evolutionary & environmental sciences

For a reference copy of the document with all sections, see nature.com/documents/nr-reporting-summary-flat.pdf

Life sciences study design

All studies must disclose on these points even when the disclosure is negative.

Sample size	n/a. This paper does not report any empirical study.
Data exclusions	n/a. CoVex is an exploratory platform and it is optional for user to exclude e.g. non-approved drugs from the analysis.
Replication	n/a. This paper does not report any empirical study.
Randomization	n/a. This paper does not report any empirical study.
Blinding	n/a. This paper does not report any empirical study.

Reporting for specific materials, systems and methods

We require information from authors about some types of materials, experimental systems and methods used in many studies. Here, indicate whether each material, system or method listed is relevant to your study. If you are not sure if a list item applies to your research, read the appropriate section before selecting a response.

Materials & experimental systems

n/a	Involved in the study	
x		Antibodies
x		Eukaryotic cell lines
x		Palaeontology and archaeology
x		Animals and other organisms
x		Human research participants
x		Clinical data
x		Dual use research of concern

Methods

n/a	Involved in the study	
x		ChIP-seq
x		Flow cytometry
x		MRI-based neuroimaging
